# Melodic Priming of Motor Sequence Performance: The Role of the Dorsal Premotor Cortex

**DOI:** 10.3389/fnins.2016.00210

**Published:** 2016-05-10

**Authors:** Marianne A. Stephan, Rachel Brown, Carlotta Lega, Virginia Penhune

**Affiliations:** ^1^Department of Psychology, Concordia UniversityMontreal, QC, Canada; ^2^International Laboratory for Brain, Music and Sound Research (BRAMS), University of MontrealMontreal, QC, Canada; ^3^Department of Neuropsychology and Psychopharmacology, Maastricht UniversityMaastricht, Netherlands; ^4^Department of Psychology, University of Milano-BicoccaMilan, Italy

**Keywords:** auditory-motor learning, dorsal premotor cortex, continuous theta burst stimulation, consolidation, crossmodal learning, transcranial magnetic stimulation, melodic priming, motor learning

## Abstract

The purpose of this study was to determine whether exposure to specific auditory sequences leads to the induction of new motor memories and to investigate the role of the dorsal premotor cortex (dPMC) in this crossmodal learning process. Fifty-two young healthy non-musicians were familiarized with the sound to key-press mapping on a computer keyboard and tested on their baseline motor performance. Each participant received subsequently either continuous theta burst stimulation (cTBS) or sham stimulation over the dPMC and was then asked to remember a 12-note melody without moving. For half of the participants, the contour of the melody memorized was congruent to a subsequently performed, but never practiced, finger movement sequence (Congruent group). For the other half, the melody memorized was incongruent to the subsequent finger movement sequence (Incongruent group). Hearing a congruent melody led to significantly faster performance of a motor sequence immediately thereafter compared to hearing an incongruent melody. In addition, cTBS speeded up motor performance in both groups, possibly by relieving motor consolidation from interference by the declarative melody memorization task. Our findings substantiate recent evidence that exposure to a movement-related tone sequence can induce specific, crossmodal encoding of a movement sequence representation. They further suggest that cTBS over the dPMC may enhance early offline procedural motor skill consolidation in cognitive states where motor consolidation would normally be disturbed by concurrent declarative memory processes. These findings may contribute to a better understanding of auditory-motor system interactions and have implications for the development of new motor rehabilitation approaches using sound and non-invasive brain stimulation as neuromodulatory tools.

## Introduction

There is emerging evidence that hearing melodic sound patterns can facilitate motor learning and memory (e.g., Lahav et al., 2005). In parallel, there is evidence for the role of the dorsal premotor cortex (dPMC) in auditory-motor integration (e.g., Chen et al., 2012). Here, we investigated whether continuous theta burst stimulation (cTBS) over the dPMC modulates auditory-motor crossmodal facilitation of motor learning.

Sensory information has been shown to influence concurrent and, most importantly, also subsequent motor performance. For example, observing an actor learning to move a robotic arm in a force field facilitated subsequent execution of the same task (Mattar and Gribble, 2005). Another study demonstrated improved timing of sequential cursor movements after previous observation of cursor movements on a computer screen (Hayes et al., 2010). Based on a study showing that action observation increased the probability of transcranial magnetic stimulation (TMS)-evoked involuntary thumb movements to fall in the observed direction, Stefan et al. (2005) proposed that observation alone (without physical training) may induce lasting specific motor memory traces similar to physical training, and concluded that the visual mirror neuron system may play a role in memory formation and human motor learning.

Consistent with evidence in the visual domain, auditory action-related stimuli can influence the motor system and several studies have demonstrated beneficial effects of sound on motor learning and memory consolidation. For example, Hommel (1996) showed that when participants learned to associate high and low tones with left and right keypresses, hearing the tones facilitated the corresponding keypress response. Moreover, simultaneously presented tones have been shown to facilitate the learning of finger movement sequences if they are mapped onto the movements in a contingent manner (in which left to right movements are associated with tones of ascending pitch; Hoffmann et al., 2001; Stöcker et al., 2003). Also, listening to a previously practiced piano piece can lead to motor performance improvements without additional physical practice (Lahav et al., 2005). In a recent study we provided the first evidence that exposure to a tone sequence alone can facilitate subsequent performance of a congruent, but never physically-practiced, motor sequence as compared to an incongruent motor sequence (Stephan et al., 2015). In this study non-musicians were asked to listen to and memorize a 12-tone melody. They were then tested on a visually-cued motor sequence learning task, where the motor sequence was either congruent or incongruent with the learned melody. Results showed that performance on the congruent sequence was enhanced compared to the incongruent sequence, demonstrating cross-modal transfer from melody listening to subsequent performance of a new, never physically practiced, motor sequence. This suggests that auditory information can prime the motor system for a subsequent motor task, paralleling previous findings in the visual domain.

Auditory-motor learning has been related to plastic neuronal changes within a network of auditory and motor brain areas (see Zatorre et al., 2007 for review). When individuals passively listen to melodies they have previously learned to perform, they engage auditory cortex, primary motor cortex (M1), PMC, and the supplementary motor area (Bangert and Altenmüller, 2003; Bangert et al., 2006; D'Ausilio et al., 2006; Baumann et al., 2007; Lahav et al., 2007; Lappe et al., 2008). Using TMS, D'Ausilio et al. (2006) found that after only 30 min of piano practice musicians showed increased intra-cortical facilitation when listening to a rehearsed piece as compared to a non-rehearsed piece. There is also evidence that motor brain responses to action-related sound can be induced even in non-musicians after relatively short periods of auditory-motor training. In an electroencephalography (EEG) study with non-musicians, 20 min of piano training with a constant key-tone mapping induced changes in auditory-sensorimotor co-activity during passive listening and silent piano playing, as compared to piano training with random key-tone assignments (Bangert and Altenmüller, 2003). Finally, work in our laboratory has demonstrated that non-musicians showed activity in motor regions, including the PMC, supplementary motor area, and cerebellum when passively listening to musical rhythms with no intent to move (Chen et al., 2008).

Within the brain network involved in fine motor control and motor learning, the PMC is suggested to be important for the selection and preparation of movements (Kurata, 1993; Hoshi and Tanji, 2007) as well as in the integration of multisensory information relevant for movement and its translation into motor plans (Gentile et al., 2011). In particular the dPMC is known for its role in linking sensory cues with a motor command. Importantly, it is hypothesized to be involved in the selection of movements based on learned associations; that is, a sensory stimulus may represent a conditional rule indicating which movement to select among competing alternatives (Passingham, 1985; Wise et al., 1996; Schluter et al., 1998; Rushworth et al., 2003; Hoshi and Tanji, 2007; Zatorre et al., 2007). In line with this suggestion, TMS over the dPMC has been shown to slow responses in choice reaction time tasks (Schluter et al., 1998; Johansen-Berg et al., 2002; Mochizuki et al., 2005). Evidence thus far suggests that the PMC is the only cortical motor brain area directly connected with the posterior superior temporal gyrus and with M1. The PMC may therefore give auditory information relatively direct access to M1, and this may underlie its role in auditory-motor integration (Zatorre et al., 2007). Accordingly, we have proposed that the PMC is important for encoding associations between sounds and actions through its connections with posterior auditory regions, and that this network may be spontaneously engaged by movement-relevant sounds (Zatorre et al., 2007; Chen et al., 2009). Consistent with this hypothesis, the dPMC was shown to be specifically engaged in learning of key press to pitch associations during short-term piano training (Chen et al., 2012). Thus, the dPMC is likely to be involved in the transformation of auditory information to motor programs, and may therefore mediate the influence of auditory information upon motor memory.

To test these ideas, the present study used cTBS to investigate the role of the dPMC in melodic priming of motor sequence performance. To do this we used the same task previously found to show melodic priming. In this task non-musicians listened to and memorized a melody which was either congruent or incongruent to a subsequently performed finger movement sequence (Stephan et al., 2015). In the current experiment, we applied cTBS or sham stimulation over the dPMC just before listening to the melody. We hypothesized that cTBS over the dPMC before melody listening would disrupt the auditory priming of motor sequences and therefore reduce facilitation of the congruent as compared to the incongruent motor sequence.

## Materials and methods

### Subjects

Fifty-two healthy, young participants without psychiatric or neurological disorders were tested [21 men, 31 women; age: mean (*M*) 23.5, standard deviation (*SD*) 4.38 years]. Participants were selected to have little musical training. They had on average 1.10 (1.34) years of formal or informal musical training [median (Mdn): 1, range: 0–5], which was stopped on average 10.0 (3.87) years before study onset (Mdn: 10, range: 2–20). They were all right-handed, according to the Edinburgh Handedness Inventory (Oldfield, 1971). All participants were screened for any contraindications to TMS (Rossi et al., 2009) and gave written informed consent prior to their inclusion in the study. One participant in the Sham group was identified as an outlier on several learning parameters based on the criterion that their score was less or greater than Q1–1.5^*^IQR or Q3+1.5^*^IQR, respectively (Q1, first quartile; Q3, third quartile; IQR, Interquartile Range) and thus excluded from analysis. The study conformed to the principles of the Declaration of Helsinki and was approved by the ethics committee “Comité d'éthique de la recherche en santé” (CERES) of the University of Montreal.

### Tasks and procedure

**Figure 1:**
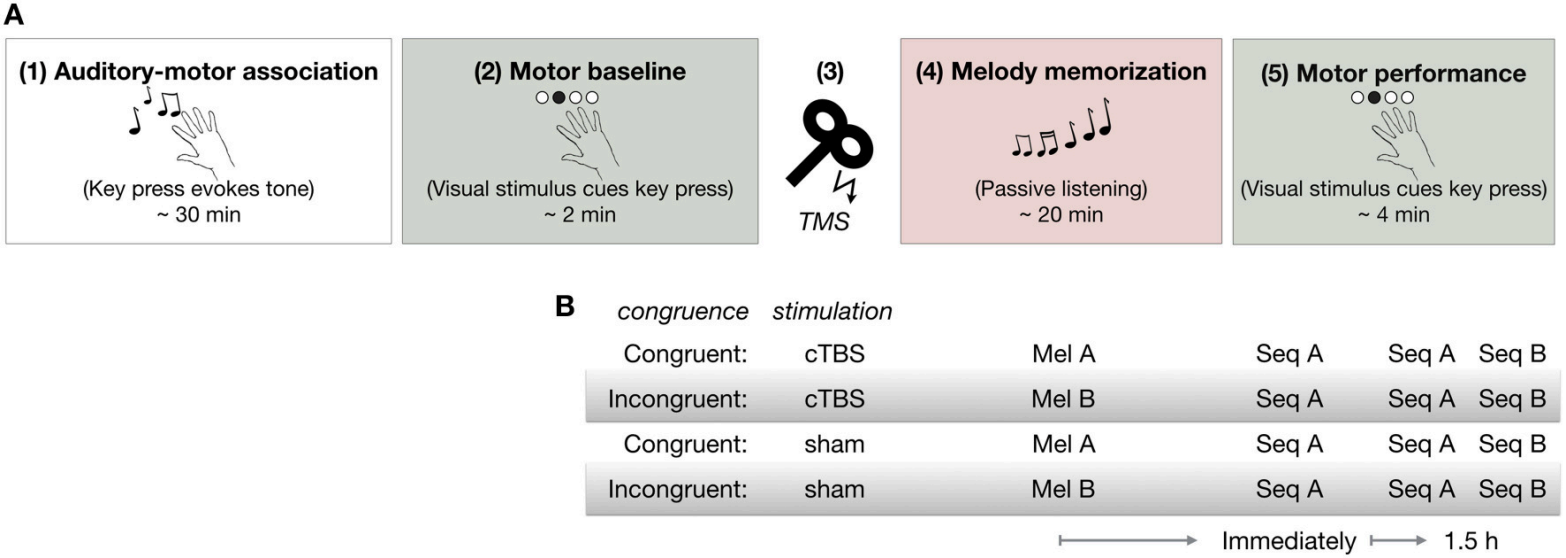
**(A)** Procedure **(B)** Experimental groups. cTBS, continuous theta burst stimulation; Mel A, melody A; Mel B, melody B; Seq A, motor Sequence A; Seq B, motor Sequence B.

As shown in Figure 1A, all participants first learned to associate each of four tones with a particular finger movement during the *Auditory-motor association* task (1) in which they performed self-selected key presses with sound feedback. Next, they performed the *Motor baseline* task (2) to establish baseline RTs to the visual cues. Participants then received either cTBS or sham stimulation over the dPMC [(3) *cTBS* over *dPMC*]. Immediately afterwards, they performed the *Melody memorization* task (4) in which they were instructed to memorize a 12-note melody composed of four tones previously trained in the Auditory-motor association task. There were two melodies, A (Mel A) and B (Mel B) which were congruent or incongruent with the pattern of finger movements in sequence A (Seq A) in the subsequent motor task (Figure 1B). All participants performed Seq A immediately after melody memorization, as well as 1.5 h later. Following performance of Seq A 1.5 h after melody memorization, all participants also performed a movement sequence B (Seq B) [(5) *Motor performance*]. There were thus four groups overall, two cTBS groups (Congruent-cTBS and Incongruent-cTBS) and two control groups (Congruent-sham and Incongruent-sham) (*n* = 13 per group, except in the sham-Congruent group in which one participant was excluded: *n* = 12).

#### (1) Auditory-motor association

To ensure that all groups had the same baseline association between the key press responses and the specific tones, participants performed a familiarization task in which they were instructed to make self-selected key-presses on four keys of a computer keyboard using the fingers of their right hand. The index, middle, ring finger, and pinky finger were pre-assigned to the v, b, n, and m keys. Each key produced a particular tone [*v* = C3 (130.81 Hz), *b* = E3 (164.81 Hz), *n* = G3 (196.00 Hz), *m* = C4 (261.63 Hz)]. Each participant performed four runs of 360 key presses each (about 30 min), with short breaks in between as needed.

To determine whether there was any difference between groups in the effect of the familiarization task, participants performed a 1-min auditory-motor facilitation test immediately before and after familiarization. In this test, assessing the effect of the tones on motor responses, participants used the same four keys to respond as quickly and as accurately as possible to visual cues appearing at one of four different horizontal positions (with the m key for the most rightward circle, the v key for the most leftward circle, etc.). Simultaneous with each visual stimulus, participants heard a tone, which was either congruent (in 50% of the trials) or incongruent with the previously primed association. There was a fixed 600 ms delay between each response and the subsequent stimulus, regardless of whether responses were correct or wrong. No feedback was given. Consistent with previous studies (see also Rusconi et al., 2005), we expected that the motor response to the visual stimulus would be facilitated by congruent sounds and slowed by incongruent sounds.

#### (2) Motor baseline

To ensure that all groups had the same baseline ability to perform the motor task, participants performed a visuo-motor response task as quickly and as accurately as possible using the same four keys and the same visual cues as before. After each key press, the subsequent cue was displayed immediately, regardless of whether the response had been correct or not. No auditory input or feedback was provided. The motor baseline task consisted of 192 quasi-randomly cued key-presses and lasted for about 2 min.

#### (3) cTBS over dPMC

In order to investigate the role of the dPMC in auditory-motor learning, participants received either cTBS or sham stimulation over the dPMC immediately following the motor baseline and before melody memorization. Details of the stimulation and coil placement are given below.

#### (4) Melody memorization

**Figure 2:**
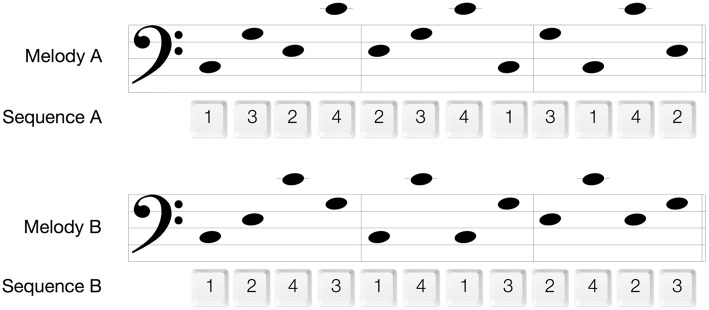
**Melodies and motor sequences**. 1, index finger; 2, middle finger; 3, ring finger; 4, pinky finger.

The aim of the melody memorization phase was to prime the motor system for a later performance of motor sequences. Participants listened to a single 12-tone melody presented 90 times in three 30-trial blocks (~20 min in total). The melody was comprised of the four movement-associated tones and either congruent (Mel A: C3-G3-E3-C4-E3-G3-C4-C3-G3-C3-C4-E3, Congruent group) or incongruent (Mel B: C3-E3-C4-G3-C3-C4-C3-G3-E3-C4-E3-G3 Incongruent group) to the subsequently performed Seq A (Figure 2). The duration of each of the melodies was 11 s and consisted of quarter notes only. In each melody the same pitch was not repeated more than once in a row and each pitch was presented three times. The transitions C3 to C4 and C4 to C3 (the largest pitch/finger distance) occurred once in each of the melodies. There was a 2-s time interval between melody presentations. Participants were told that they should try to remember this melody, since they would be asked later on to write it down and to hum it. During the memorization sessions, they were instructed to relax and to refrain from movement. This was monitored closely by visual observation and surface electromyography (EMG) from the first dorsal interosseus muscle (FDI, see below). All sounds and melodies were synthesized with the “GarageBand” music editing software (GarageBand 6.0.4, Apple Inc. 2011) and had a synthesized piano timber.

In order to test whether all groups were equally able to memorize the melody, explicit knowledge of the melody was assessed after each of the three memorization blocks using the methods developed in Stephan et al. (2015). First, participants were asked to hum the melody into a microphone, which was recorded for later off-line analysis. Secondly, participants were instructed to write the melody using a simplified notation scheme consisting of drawing circles on a grid with four horizontal lines. In order to determine whether any participant used a memorizing strategy related to finger movements, participants had to indicate on a questionnaire the strategy used to memorize the melody. This was done once, after the first memorizing block.

#### (5) Motor performance

In order to assess the effect of melody memorization on motor performance, participants performed a visuo-motor response task using the same four keys and the same visual cues as during the baseline motor performance task. They were instructed to press keys with their right hand as quickly and as accurately as possible in response to a repeating sequence of visual stimuli appearing on a computer screen. The 12-key-press sequence, referred to as Seq A (Figure 2), was repeated 30 times (lasting about 4 min) and was performed immediately as well as 1.5 h after melody memorization (Figure 1B). Following the second assessment 1.5 h after melody memorization all participants also performed motor Seq B. (Seq B was congruent with Mel B, which was only memorized by the Incongruent groups.) No auditory input or feedback was provided.

In order to check whether there were any differences in explicit knowledge of the motor sequence between groups, explicit knowledge was assessed after the final motor test at the end of the experiment. Participants were asked to write out the sequence of finger movements using a number to represent each finger (index finger: 1; middle finger: 2; ring finger: 3; pinky finger: 4).

### Transcranial magnetic stimulation

**Figure 3:**
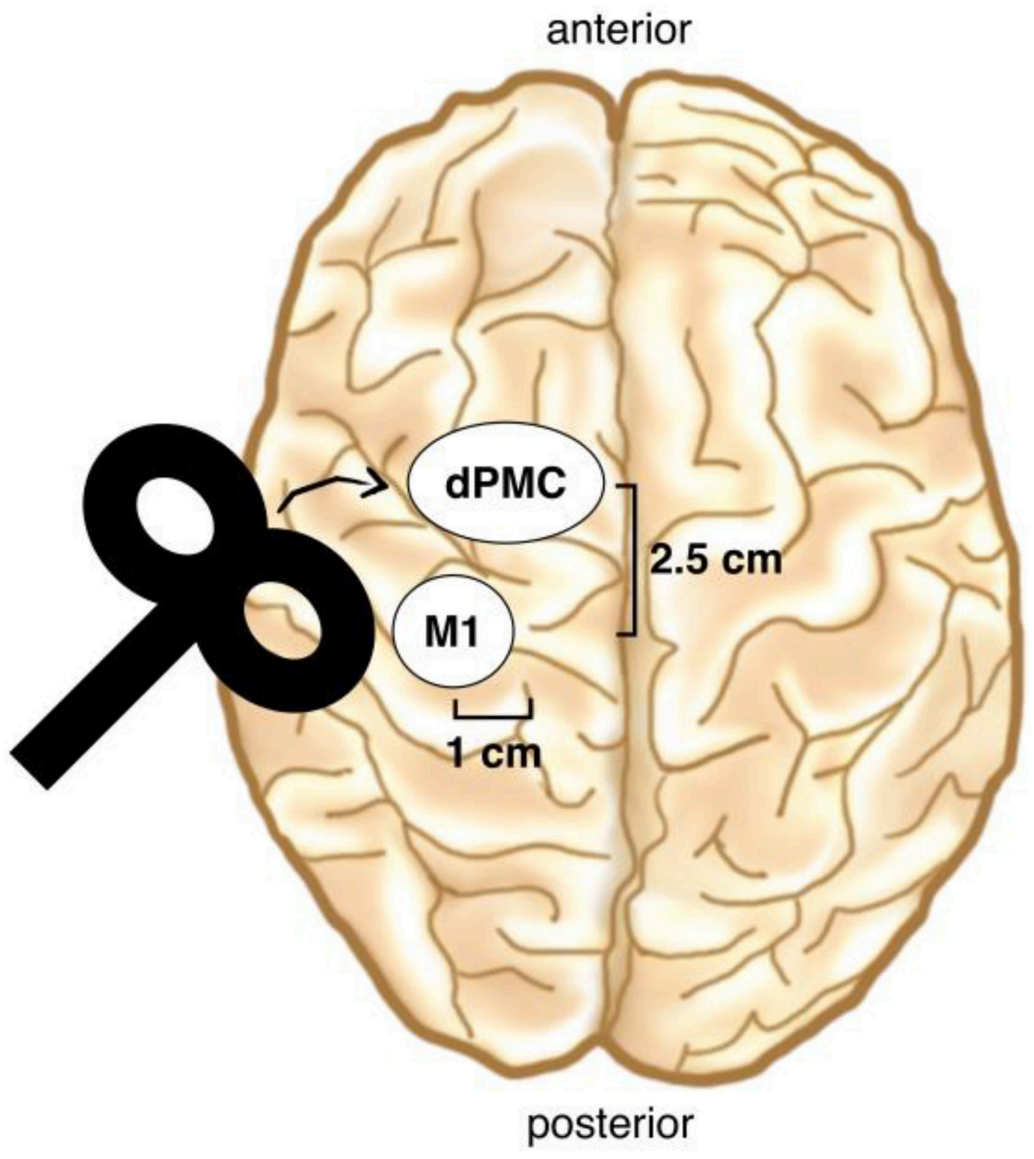
**TMS coil placement**. The dPMC was located 1 cm medial and 2.5 cm anterior of the motor “hot-spot” (M1). (Brain drawing from Sandrine Muller).

Immediately before the melody memorization session, each participant underwent either cTBS or sham stimulation over the left dPMC. Continuous TBS was applied at 80% of the active Motor Threshold (aMT) and consisted of a continuous 40-s train of TBS with 600 stimuli [short bursts of three stimuli at 50 Hz which were repeated at a rate in the theta range (5 Hz)] (Hellriegel et al., 2012; Platz et al., 2012). The dPMC was located 1 cm medial and 2.5 cm anterior at the same laterality as the motor “hot-spot” (Figure 3; Ortu et al., 2009), defined as the site where the largest motor evoked potentials (MEPs) could be evoked in the relaxed right FDI muscle. We checked in each participant whether stimulation over the defined dPMC evoked any MEPs and moved the coil 0.5 cm anterior in three subjects where this was the case (Bestmann et al., 2005). TMS was applied trough a 70 mm figure-of-eight coil, using a Super Rapid Biphasic Stimulator (Magstim, Whitland, UK) with the handle pointing 45° postero-laterally away from the midline for both M1 and the dPMC. A TMS neuronavigation system (Brainsight 2; Rogue Research Inc., Canada) was used to ensure a constant coil position during cTBS. The aMT was determined according to standard procedure during slight tonic contraction of the right FDI muscle (20% of maximal force), using the software based “adaptive method” developed by (Awiszus, 2003) (Motor Threshold Assessment Tool (MTAT, version 2.0: http://www.clinicalresearcher.org/software.htm). An MEP ≥ 200 μV peak-to-peak amplitude was feed back to the software as valid response. EMG recordings were obtained from the right FDI muscle, with conventional surface electrodes in a belly-tendon montage. Signals were amplified, bandpass filtered (1 Hz–2 kHz) and sampled at a rate of 10 kHz.

### Analysis

The first key press time was discarded in the auditory-motor association, motor baseline, and motor performance test, as it was sometimes very prolonged. Moreover, RTs less or greater than Q1–1.5^*^IQR or Q3+1.5^*^IQR, respectively, were excluded. Statistical analyses were performed with R (R Core Team, 2014). For all outcome measures, normal distribution and homogeneity of variances were tested. When appropriate, non-parametric permutation-based analogs of the mixed factorial ANOVAs [R package “ez,” Lawrence (2013)] and Wilcoxon rank sum tests were performed. (The permutation-based tests confirmed the ANOVA results reported below).

#### Auditory-motor association

In order to analyze baseline auditory-motor facilitation, the change in error rate (the percentage of false responses) from before to after the auditory-motor association phase was calculated for incongruent trials (delta InC) and congruent trials (delta C). Delta C was then subtracted from delta InC to get a measure of the relative increase in errors in incongruent trials when compared to congruent trials for each participant. The same measure was calculated for the mean RT. Two mixed factorial ANOVAs were performed with the relative increase in errors and the relative increase in RT as the dependent variables, and congruence (Congruent, Incongruent group) and stimulation (cTBS, sham) as the between-subject factors (Pinheiro et al., 2014)). During the auditory-motor association task, participants pressed all four keys roughly the same number of times, as revealed by histograms of key-presses for each participant.

#### Motor performance

To assess motor performance, the mean RT of correct responses was calculated for each subject and session. As there was no difference in motor performance between congruence or stimulation conditions at baseline [congruence: *F*_(1, 47)_ = 0.89, *p* = 0.35, generalized eta squared effect size ($\eta_{G}^{2}$) = 0.019; stimulation: *F*_(1, 47)_ = 0.076, *p* = 0.78, $\eta_{G}^{2}$ = 0.002; stimulation × congruence: *F*_(1, 47)_ = 0.15, *p* = 0.70, $\eta_{G}^{2}$ = 0.003], mean baseline RTs were subtracted from the mean RTs immediately and 1.5 h after melody memorization. These normalized RTs were then used for data analysis.

To determine the immediate effect of melody memorization and cTBS on motor performance and to investigate whether the effect would still be present during early offline consolidation 1.5 h later, a mixed factorial ANOVA was performed with the RT as the dependent variable, congruence (Congruent, Incongruent) and stimulation (cTBS, sham) as the between-subject factors, and session (Seq A immediately and Seq A 1.5 h after melody memorization) as the within subject factor. Another mixed factorial ANOVA for the RTs 1.5 h after melody memorization was performed, with congruence (Congruent, Incongruent) and stimulation (cTBS, sham) as between-subject factors and sequence (A, B) as the within-subject factor.

#### Explicit knowledge of the melody

Recordings of participants singing back the melodies were scored off-line by counting the longest sequence of tones with the correct contour (considering only relative changes in pitch, e.g., if the tone was higher or lower, ignoring mistakes in absolute pitch intervals). The score for the written melody was calculated by counting the longest sequence of written tones with the correct contour when considering only relative changes in pitch. The mean of the third administration of the hummed and written melody memorization assessments was used as the measure of participants' explicit knowledge of the melody. A mixed factorial ANOVA was performed with the melody memory score as the dependent variable, and congruence (Congruent, Incongruent) and stimulation (cTBS, sham) as the between-subject factors.

#### Explicit knowledge of the motor sequence

Explicit knowledge of the motor sequence was determined by counting the length of the longest correctly reported sequence. A mixed factorial ANOVA was performed with the motor sequence memory score as the dependent variable, and congruence (Congruent, Incongruent) and stimulation (cTBS, sham) as the between-subject factors.

## Results

### Auditory-motor association

There was no difference in auditory-motor facilitation between congruence or stimulation conditions at baseline (relative increase in RT: congruence: *F*_(1, 47)_ = 0.12, *p* = 0.73, $\eta_{G}^{2}$ = 0.002; stimulation: *F*_(1, 47)_ = 0.26, *p* = 0.62, $\eta_{G}^{2}$ = 0.006; congruence × stimulation: *F*_(1, 47)_ = 0.45, *p* = 0.50, $\eta_{G}^{2}$ = 0.01; relative increase in errors: congruence: *F*_(1, 47)_ = 0.21, *p* = 0.65, $\eta_{G}^{2}$ = 0.004; stimulation: *F*_(1, 47)_ = 0.021, *p* = 0.88, $\eta_{G}^{2}$ = 0.0004; congruence × stimulation: *F*_(1, 47)_ = 0.084, *p* = 0.77, $\eta_{G}^{2}$ = 0.002).

### Motor performance

**Figure 4:**
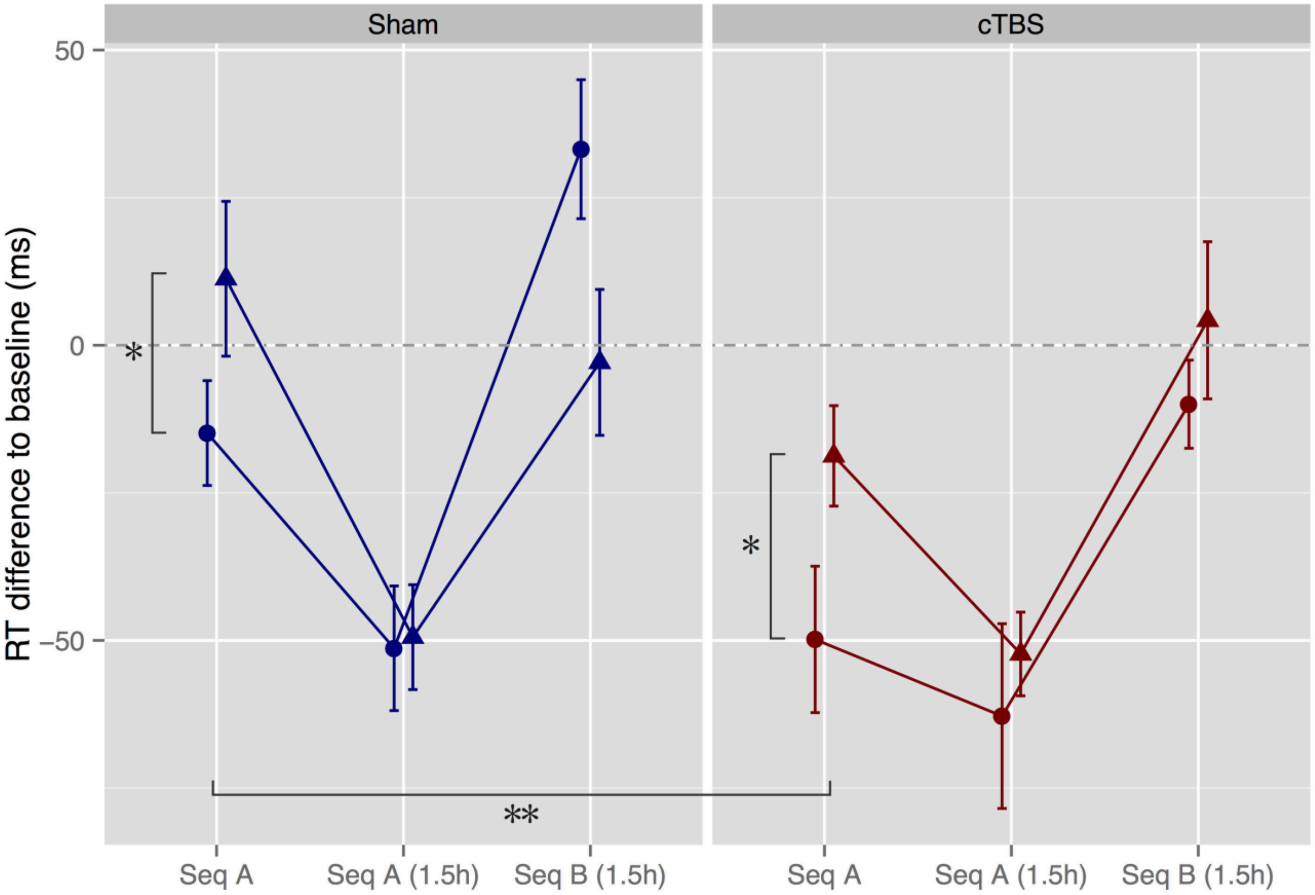
**Motor performance tests immediately after melody memorization as well as after a 1.5h delay**. Hearing Melody A led to significantly faster performance of motor Sequence A (Seq A) immediately thereafter compared to hearing the incongruent Melody B (^*^p < 0.05). In addition, cTBS speeded up motor performance in both the congruent and the incongruent groups (^**^p < 0.01). Triangles, Incongruent groups. Circles, Congruent groups. Error bars, ± standard error of the mean.

To assess the effect of melody memorization on motor performance, we compared normalized RTs across groups for the immediate and 1.5 h tests. For the immediate test, results showed a main effect of congruence, where, as expected, RTs were significantly shorter in the Congruent condition than in the Incongruent condition [*F*_(1, 47)_ = 6.79, *p* = 0.012, $\eta_{G}^{2}$ = 0.13], replicating previous results (Stephan et al., 2015). In addition, there was a significant main effect of stimulation, indicating that participants who had undergone cTBS were significantly faster than participants who had undergone sham stimulation [*F*_(1, 47)_ = 9.00, *p* = 0.004, $\eta_{G}^{2}$ = 0.16] (Figure 4).

From the immediate to the 1.5 h test, there was a significant main effect of session [*F*_(1, 47)_ = 81.2, *p* < 0.0001, $\eta_{G}^{2}$ = 0.18], as well as significant session × congruence [*F*_(1, 47)_ = 7.87, *p* = 0.007, $\eta_{G}^{2}$ = 0.021] and session × stimulation interactions [*F*_(1, 47)_ = 10.5, *p* = 0.002, $\eta_{G}^{2}$ = 0.027]. These findings indicate that all groups improved between the immediate and 1.5 h test sessions, but that the Incongruent and sham groups showed greater improvements, such that there was no significant effect any more of stimulation or congruence for the 1.5 h session [stimulation: *F*_(1, 47)_ = 0.43, *p* = 0.52, $\eta_{G}^{2}$ = 0.009; congruence: *F*_(1, 47)_ = 0.32, *p* = 0.57, $\eta_{G}^{2}$ = 0.007; stimulation × congruence: *F*_(1, 47)_ = 0.15, *p* = 0.70, $\eta_{G}^{2}$ = 0.003].

To assess whether Seq A was learned (determined by a significant increase in RT from Seq A to Seq B) and whether memory traces of Seq B in the Incongruent group were still present 1.5 h after melody memorization (determined by an interaction sequence × congruence) we compared performance of Seq A from the 1.5 h session with performance of Seq B immediately following. Results showed a significant main effect of sequence, such that all groups showed increases in RT when switching from Seq A to Seq B [*F*_(1, 47)_ = 75.9, *p* < 0.0001, $\eta_{G}^{2}$ = 0.37]. There were no other significant main effects or interactions [stimulation × sequence × congruence: *F*_(1, 47)_ = 2.32, *p* = 0.13, $\eta_{G}^{2}$ = 0.018].

To further explore the apparent between-group difference for Seq B (see Figure 4), separate ANOVAs for participants in the sham and cTBS conditions were performed. For participants in the sham condition, results revealed again a significant main effect of sequence [*F*_(1, 23)_ = 68.6, *p* < 0.0001, $\eta_{G}^{2}$ = 0.43] and, as hypothesized, an interaction sequence × congruence [*F*_(1, 23)_ = 5.90, *p* = 0.023, $\eta_{G}^{2}$ = 0.061), due to the fact that the Incongruent group performed Seq B faster than the Congruent group (Wilcoxon rank sum test: *W* = 114, *Z* = 1.96, *p* = 0.052, *r* = 0.39) while there was no difference between groups in performance of Seq A (*W* = 76, *Z* = 0.11, *p* = 0.94, *r* = 0.022). In contrast, participants who had undergone cTBS showed as well a significant effect of sequence [*F*_(1, 24)_ = 24.2, *p* = 0.0001, $\eta_{G}^{2}$ = 0.32] but no interaction sequence × congruence [*F*_(1, 24)_ = 0.028, *p* = 0.87, $\eta_{G}^{2}$ = 0.0005], as RTs in the Congruent condition were facilitated and as low as the RTs in the Incongruent condition (Seq B: *W* = 73, *Z* = 0.59, *p* = 0.58, *r* = 0.12; Seq A: *W* = 88, *Z* = 0.18, *p* = 0.88, *r* = 0.035, see Figure 1).

The average percentage of errors across all motor performance tests was very low, 3.18% (*SD* 2.43, Mdn 2.62) for participants who underwent cTBS and 3.05% (*SD* 2.83, Mdn 2.23) for the sham group. There was no effect of the stimulation [*F*_(1, 47)_ = 0.045, *p* = 0.83, $\eta_{G}^{2}$ = 0.0008] or the congruence on the percentage of errors [*F*_(1, 47)_ = 0.55, *p* = 0.46, $\eta_{G}^{2}$ = 0.012, stimulation × congruence: *F*_(1, 47)_ = 0.092, *p* = 0.76, $\eta_{G}^{2}$ = 0.002]. These findings were thus not analyzed any further.

### Explicit knowledge of the melody

There was no effect of stimulation or congruence on the ability to memorize the melody [stimulation: *F*_(1, 47)_ = 0.20, *p* = 0.66, $\eta_{G}^{2}$ = 0.005; congruence: *F*_(1, 47)_ = 0.96, *p* = 0.33, $\eta_{G}^{2}$ = 0.020; stimulation × congruence: *F*_(1, 47)_ = 0.80, *p* = 0.38, $\eta_{G}^{2}$ = 0.017]. The length of the longest correctly reported sequence of tones was on average 8.87 (*SD* 2.66) out of a total of 12 tones. Out of the 51 included participants only two used a memorizing strategy related to finger movements (imagined playing the keys, one participant in the Congruent-cTBS, and one participant in the Congruent-sham group), indicating that the majority of participants were not engaged in motor imagery.

### Explicit knowledge of the motor sequence

There was no effect of stimulation or congruence on explicit knowledge for motor Seq A tested 1.5 h after melody memorization [stimulation: *F*_(1, 47)_ = 0.047, *p* = 0.83, $\eta_{G}^{2}$ = 0.001; congruence: *F*_(1, 47)_ = 0.25, *p* = 0.62, $\eta_{G}^{2}$ = 0.005; stimulation × congruence: *F*_(1, 47)_ = 1.37, *p* = 0.25, $\eta_{G}^{2}$ = 0.028]. The length of the longest correctly reported Seq A was on average 5.63 (*SD* 2.96) out of a total of 12 finger movements. For motor Seq B, a trend for a better explicit knowledge in the Incongruent group compared to the Congruent group was detected [congruence: *F*_(1, 47)_ = 3.24, *p* = 0.078, $\eta_{G}^{2}$ = 0.065; Incongruent: *M* 7.73, *SD* 3.67, Congruent: *M* 5.96, *SD* 3.17], but as for Seq A, no effect of stimulation [*F*_(1, 47)_ = 1.01, *p* = 0.32, $\eta_{G}^{2}$ = 0.019] and no interaction stimulation × congruence [*F*_(1, 47)_ = 0.004, *p* = 0.95, $\eta_{G}^{2}$ = 0.00008]. The length of the longest correctly reported Seq B was on average 6.86 (*SD* 3.52) out of a total of 12 finger movements.

## Discussion

Our results show that exposure to a movement-related tone sequence can crossmodally and specifically affect subsequent performance of a motor sequence that has never been physically-practiced. Importantly, this phenomenon was not impaired by cTBS over the dPMC. Instead, our data provides evidence that cTBS over the dPMC applied directly after the motor baseline task and immediately before melody memorization enhanced offline consolidation of the procedural motor skill learned in the baseline task. We hypothesize that cTBS may have removed interference between procedural motor skill consolidation and the declarative melody memorization task. Moreover, cTBS may have facilitated switching from Seq A to the new Seq B 1.5 h after melody memorization in the Congruent group who had never listened to Mel B before. We can only speculate that this may have been due to a more flexible auditory-motor association that could be applied to Seq B. Finally, findings from the Sham control group show that motor memory traces induced by memorization of Mel B are still present 1.5 h after learning, despite intermediate practice on Seq A.

### Effect of melody listening on immediate motor performance

In this study, listening to and memorizing a melody lead to improved performance of a congruent finger movement sequence as compared to listening to an incongruent melody. This is consistent with our previous work and provides further evidence that exposure to a series of tones that have previously been associated with movements can facilitate performance of a never physically-practiced motor sequence (Stephan et al., 2015). The current findings are also in line with studies from the visual domain, in which observation can improve physical execution of the observed motor tasks (Mattar and Gribble, 2005; Hayes et al., 2010), or where observation increased the probability of TMS-evoked involuntary movements in the observed direction (Stefan et al., 2005). The current findings therefore add further support to the idea that perception of movement-relevant stimuli, either through visual observation or auditory priming, can induce specific motor memory traces similar to physical training.

Sensory priming of movement may result from planning and prediction mechanisms of the human motor system, particularly the PMC. Action planning has been suggested to involve the anticipation of direct “proximal” sensory consequences of movement, such as kinesthetic or tactile experiences, as well as the anticipation of more indirect “distal” effects, such as a light or a tone evoked by a finger movement (Hommel, 2005). However, in order to optimally interact with our continuously changing environment we have to anticipate not only single events, but most often ongoing sequences of events (Schubotz and von Cramon, 2002). Accordingly, it has been hypothesized that the PMC is involved in predicting sequences of sensory stimuli whenever they are relevant for the motor system (Schubotz and von Cramon, 2002, 2003; Schubotz, 2007). Based on this hypothesis, even arbitrary movement-relevant stimulus sequences, such as melodies, may be transformed into motor representations in the PMC whenever their sequential pattern has to be analyzed. Thus, in the current study memorizing a movement-related tone sequence would lead to the induction of a feed-forward motor memory representation that could facilitate motor performance.

### Effect of cTBS over the dPMC on immediate auditory-motor learning

Unexpectedly, cTBS over the dPMC did not impair the effect of melody memorization on immediate motor performance, since after sham as well as after cTBS, participants in the Congruent group performed better than participants in the Incongruent group. Interestingly, this finding is compatible with recent evidence from animal literature indicating considerable robustness of neural representations that drive specific future movements after silencing PMC in one hemisphere (Li et al., 2016). The authors suggested that cortical networks maintaining motor representations are organized in a redundant fashion, allowing the unperturbed hemisphere to restore preparatory activity in the opposite hemisphere. It is conceivable that a similar compensatory mechanism may also have taken place in the current study. In addition, for both the Congruent and Incongruent groups, cTBS lead to faster RTs in the immediate motor performance test.

We hypothesize that cTBS over the dPMC, applied after the random motor baseline task and before melody memorization, enhanced offline procedural motor skill consolidation of the baseline task. The reason for this observed effect might be that cTBS reduced interference of melody memorization on consolidation of the motor baseline task. Consolidation can be defined as a process where memory becomes enhanced, reflected in an “off-line” improvement in task performance, or, where memory becomes less susceptible to interference (Robertson et al., 2004; Robertson, 2009). Consolidation of procedural (i.e., skills) and declarative (i.e., facts) memories have been shown to reciprocally interact. In particular, off-line motor skill improvement was suggested to be disrupted by subsequent declarative learning (Brown and Robertson, 2007a,b; Galea et al., 2010). It has recently been suggested that such memory interference may arise from brain areas supporting the interaction between otherwise independent memory formation processes (Robertson, 2012). For example, in a study by Cohen and Robertson (2011), inhibitory 1 Hz rTMS over M1 was suggested to benefit motor memory consolidation by overcoming interference between motor learning and subsequent declarative word list learning. It was suggested that there is a communication between motor and cognitive memory formation processes, which, when disrupted, reduces mutual interference without directly affecting the memories in isolation. This is in line with the finding of the current study in which cTBS over the dPMC enhanced motor off-line consolidation while not affecting participants' ability to memorize a melody.

Possible limitations of the study are that localizing the dPMC stimulation site relative to the M1 hot-spot (2.5 cm anterior and 1 cm medial to M1) may not be as precise as individual MRI-guided localization. Moreover, we cannot exclude the possibility that cTBS influenced motor performance via an inhibition of the dPMC or of the connected M1 during the immediate motor test. However, previous studies on the PMC or M1 using cTBS and other interfering TMS protocols shortly before or during behavioral tasks demonstrated slowed down RTs or a depressed boost in performance (Schluter et al., 1998; Johansen-Berg et al., 2002; Mochizuki et al., 2005; Hotermans et al., 2008; Gorbet and Staines, 2011). We thus consider a direct effect on the motor performance test as rather unlikely since we would have expected a slowing or at least no change rather than a speeding of RTs.

Finally, our results are also in line with the frequent observation that the direction of behavioral effects of non-invasive brain stimulation do not necessarily match the direction of presumed cortical excitability changes. Even though non-invasive inhibitory brain stimulation protocols such as 1 Hz rTMS or cTBS are usually assumed to induce a lasting “disruption” of neuronal processing in the stimulated cortical area (e.g., Huang et al., 2009), stimulation effects are complex and may vary depending on the functional state of the cortical area at the time of stimulation (Fricke et al., 2011; Fung and Robinson, 2014). For example, in a study by Pavlova et al. (2014), anodal *or* cathodal tDCS over the premotor cortex improved performance in a dexterity-demanding motor task, depending on perceived task difficulty, and specific performance features. In addition, behavioral effects of TMS may include the consequences of neuronal changes in remote interconnected brain areas rather than of changes in the stimulated region alone (Ward et al., 2010; Song et al., 2011).

### Effect of melody listening and cTBS on delayed motor performance

When comparing performance on Seq A after the 1.5 h delay to melody memorization, all groups showed gains in performance, with no significant differences between groups. This was likely due to the additional practice of Seq A at the immediate test, which allowed the Incongruent groups to catch up in motor performance.

When comparing performance on Seq B after the delay, two intriguing results appeared. First, participants in the Sham control group who had memorized Mel B performed significantly better on Seq B than participants who had memorized Mel A. This shows that motor memory traces induced by memorization of Mel B are still present 1.5 h after learning, and that they are robust to interference from performance of Seq A during the immediate and delayed test conditions. Second, the Congruent group who had memorized Mel A, but received cTBS over PMC performed better on Seq B than the Sham control group and at a similar level to the Incongruent groups who had memorized Mel B. This shows that cTBS over PMC for those who had memorized Mel A resulted in enhanced performance of the novel Seq B compared to Sham. We can only speculate that cTBS over PMC may have reduced the cost for switching from Seq A to Seq B, for example by producing a more flexible auditory-motor association that could be applied to Seq B.

## Conclusion

We conclude that memorizing a melody whose tones have been associated with movement can trigger the formation of a movement sequence representation that facilitates later performance without physical practice. We hypothesize that this is the result of feed-forward models generated by the motor system that make predictions based on sensory information that has been previously linked with a motor response, even if that link is abstract. We further suggest that cTBS over the dPMC may enhance early offline procedural motor skill consolidation in cognitive states where motor consolidation would normally be disturbed by concurrent declarative memory processes.

## Author contributions

MS designed the study, acquired, analyzed and interpreted data, and drafted and revised the work. RB designed the study, acquired data, and revised the work. CL acquired data and revised the work. VP designed the study, analyzed and interpreted data, and revised the work.

## Funding

This research was supported by the Swiss Foundation for Grants in Biology and Medicine (SFGBM) and the Swiss National Science Foundation (SNSF) to MS, the National Science and Engineering Research Council of Canada to VP (2015-04225).

### Conflict of interest statement

The authors declare that the research was conducted in the absence of any commercial or financial relationships that could be construed as a potential conflict of interest.
